# Analysis of sleep spindles in children with Asperger’s syndrome

**DOI:** 10.5935/1984-0063.20200059

**Published:** 2021

**Authors:** Rodolfo Cebreros-Paniagua, Fructuoso Ayala-Guerrero, Erik Leonardo Mateos-Salgado, Christopher Isaac Villamar-Flores, Carlos Alberto Gutiérrez-Chávez, Ulises Jiménez-Correa

**Affiliations:** 1 National Autonomous University of Mexico, Psychology Faculty - Mexico City - Mexico.; 2 Autonomous University of the State of Mexico, Psychology Faculty - Toluca - State of Mexico - Mexico.; 3 National Autonomous University of Mexico, Sleep Disorders Clinic, Medicine Faculty, Research Division - Mexico City - Mexico.; 4 National Autonomous University of Mexico, Postgraduate Program in Behavioral Neuroscience, Psychology Faculty - Mexico City - Mexico.

**Keywords:** Sleep Spindle, Asperger’s Syndrome, Neurodevelopmental Disorders

## Abstract

Sleep spindles are an element of the sleep microstructure observed on the EEG during the NREM sleep phase. Sleep spindles are associated to sleep stability functions as well as memory consolidation and optimization of different cognitive processes. On the other hand, Asperger’s syndrome (AS) is a generalized developmental disorder in which cognitive and sleep disturbances have been described. In this study we analyzed different characteristics of sleep spindles in a group of children with AS and compared them with sleep spindles of a group of children with typical development paired by age; both groups ranged from 6 to 12 years of age and were all male. We observed a statistically significant decrease in sleep spindles intrinsic frequency in different brain regions in the AS group in relation to the typical development group. This finding could be due to immaturity in brain regions related to the integration of sleep spindles; and this immaturity could be related with cognitive aspects in these patients.

## INTRODUCTION

Sleep is a highly complex phenomenon divided into different stages depending on changes in electrical brain activity (EEG). The electroencephalographic graph-elements that characterize each of the sleep stages make up the sleep microstructure, where the sleep spindles are present^1^.

Sleep spindles are integrated by waxing and waning rhythmic waves of 9 to 16Hz with duration from 0.5 to 2.5 seconds observed in frontal, central and parietal EEG derivations; these sleep microelements are observed across the N2 and N3 sleep phases. They originate in the thalamic reticular nucleus when hyperpolarization occurs due to a decrease in noradrenergic and serotonergic activity; reticular nuclei then generate electrical bursts that are transmitted to the cerebral cortex through thalamic-cortical interactions, producing inter-regional synchronization patterns^2,3^.

Sleep spindles appear between six weeks and three months of age; as age increases, both quantity and intrinsic frequency of sleep spindles increment, while their duration tends to decrease. At around 12 months of age the first form of a mature spindle can be seen, with the appearance of slow spindles on anterior regions of the brain^4^. During puberty, the spindles observed in frontal areas tend to become slower (<12Hz) compared to those of central and parietal areas where spindles commonly have a faster frequency (>12Hz). Amplitude begins to decrease and a greater decrement is observed in posterior areas of the brain. At the end of adulthood, the number of spindles begins to decrease gradually^5^.

Sleep spindles have been considered as a sleep stabilizer. This graph-element has a protective function by modulating the degree of sensory stimulation that reaches the thalamus; this is supported by clinical observations where people with hypersomnia have a higher density of spindles in relation to people with normal sleep patterns^6^. It is also observed that people with a higher density of sleep spindles have greater tolerance to presence of noise and therefore tend to have fewer awakenings than people with fewer spindles^7^. Recent studies in which sleep spindles are evoked artificially in mice show that there is a positive correlation between the amount of sleep spindles and length of NREM^8^.

Sleep spindles are also related to memory consolidation. Different researches show that there is an increase in density and power of sleep spindles in people who sleep after performing verbal and visuospatial learning, and memorization tasks, as well as motor tasks^9^. With the use of EEG and functional magnetic resonance imaging (fRMN), researchers observed a reactivation of brain areas that were used during the learning process in the subsequent sleep periods^10^. Likewise, this graph-element is associated with brain plasticity and IQ^11^.

Different studies have determined that the quantity and morphology of sleep spindles are affected in different neuropsychiatric diseases such as schizophrenia, Down syndrome and Alzheimer’s disease^12-14^. Generalized developmental disorders, which include the autistic spectrum, are a group of neurological disorders in which analysis of sleep microstructure has not been fully studied. In this context, analyzing the characteristics of sleep spindles in these patients can provide important information about sleep stability and the level of impairment of cognitive abilities.

Asperger’s syndrome (AS) is a generalized developmental disorder characterized by deficiencies in social interaction, presence of inadequate communication skills, restricted interests and stereotypical and repetitive behaviors. Cognitive and linguistic development are not delayed, as in other autism spectrum disorders^15^. Neuroanatomical studies have evidence that Asperger’s patients show a decrease in the gray and white matter density in multiple regions such as amygdala, hippocampus, prefrontal lobes, medial-frontal gyrus, right cerebellum, limbic system, parietal lobe, left thalamus, and putamen. Functional abnormalities are also observed in cerebellar connections, in the frontal and temporal cortex, as well as in the limbic system, including the amygdala and hippocampus^16,17^.

Regarding their cognitive characteristics, using different validated psychometric tests, it is observed that patients with AS present difficulties in identification of symbols and increased reaction time when performing tasks. On the contrary, a better performance is observed in arithmetic, verbal and fluency of reasoning tasks. These patients also present deficits on identification of prosody and rhythm of language. Among other cognitive alterations found in AS are social skills, executive functions, sustained attention, and coherence of thoughts^16^.

AS patients also present deficits in tasks that involve autobiographical and episodic information^18^. On the other hand, they perform well on semantic memory tasks related to remembering pairs of words, while they have difficulties with tasks related to working memory^19^.

Several studies show that patients with generalized developmental disorders, including Asperger’s syndrome, have sleep disturbances. Using questionnaires and actigraphy, we can observe difficulties in initiating and maintaining sleep, shorter total sleep time, morning awakenings and parasomnias^20-23^. However, polysomnographic recordings show contrasting results; in some cases, a decrease in total sleep time is observed and in other cases there are no differences in sleep macrostructure^24-27^.

Recent studies on sleep microstructure in patients within the autistic spectrum show a decrease in sleep spindle’s density in central regions, lower density of sleep spindles in the N2 phase in prefrontal regions and shorter duration spindles in frontal regions^28,29^. In other studies, no significant differences were observed in spindle’s density and main characteristics^30^. These studies analyze the sleep spindles in patients along the entire autistic spectrum, which includes different disorders like classic autism and AS, which may explain the inconsistencies in the results. Therefore, the objective of this study is to provide additional information about the sleep spindles characteristics exhibited during sleep in children with AS.

## MATERIAL AND METHODS

### Study design

Cross-sectional case-control study.

#### Sample description

The studied sample consisted of nine male children diagnosed with AS and nine healthy participants (HP) with age and sex matched (between 6 and 12 years).

### Participants

#### AS Group

Inclusion criteria: having a diagnosis of AS made by multidisciplinary specialists in “Caritas de Amistad” association, based on the DSM-IV TR^31^ and conducted through interviews with children and their parents.

Exclusion criteria: present evidence of sleep apnea or parasomnia during the first night of study or previously identified; also any neurological disease or consumption of hypnotics.

#### HP Group

Inclusion criteria: having age and sex mentioned previously.

Exclusion criteria: present evidence of sleep disorder during the first night of study or previously identified, consumption of medication at the time of the study and diagnosis of a chronic disease or health problem that affects sleep.

Sampling for AS group was carried out through the voluntary participation of members of “Caritas de Amistad”, an association that specializes in diagnosing and treating children and adolescents with Asperger’s syndrome. While for the HP group, convenience sampling was carried out in which relatives of members of the university community were identified and selected; the children’s parents were given a medical history to assess the health status of the candidate.

### Ethics consideration

Written informed consent was signed by each parent or guardian of all the participants. This protocol has been approved by the Ethics Committee of the Faculty of Psychology at National Autonomous University of Mexico.

### Procedure

Data collection was carried out through two polysomnographic (PSG) recordings on consecutive nights, the first one was considered for habituation to recording conditions and the second for analyzing and comparing the sleep characteristics displayed by the two groups. The PSGs were carried out in the Neuroscience Laboratory of the Faculty of Psychology at National Autonomous University of Mexico (Universidad Nacional Autónoma de México UNAM). During eight-hour studies, electrical brain (EEG), ocular (EOG), muscle (EMG), and cardiac (EKG) activities were obtained. In addition, respiratory and pulse oximetry sensors were placed. Recordings were done by a 32-channel Easy II equipment and software from Cadwell Laboratories from 2006.

During the first night, electrodes for EEG recordings were placed in C3, C4, O1 and O2 derivations. During the second night of recording, EEG electrodes were placed in F3, F4, C3, C4, T3, T4, P3, P4, O1, and O2 leads.

EEG data was obtained from monopolar leads using wave filters from 35Hz to 0.35Hz and a sensitivity of 10µV/mm^34^.

### Data analysis

The sleep phases and sleep spindles were visually identified according to the Manual of the American Academy of Sleep Medicine^32^. Sleep spindles index (number of spindles per hour of sleep), duration, amplitude and intrinsic frequency (number of waves per second) were analyzed to determine differences between both groups of participants. For the analysis of sleep spindle’s index, we identify each spindle visually in at least one of the frontal and parietal derivations.

In order to calculate duration, amplitude and frequency, 200 sleep spindles of four different EEG derivations (F3, F4, P3 and P4) were visually selected for each participant and subsequently analyzed using the Brain Storm EEG software. This resulted in 50 sleep spindles per derivation^33^; for each EEG lead, sleep spindles not associated with K complexes or arousals were visually identified independently; these spindles were selected in epochs scored as N2. For the intrinsic frequency of each spindle was obtained using the Fourier transform via the software and the results were averaged.

### Statistical analysis

We performed Mann-Whitney U test for independent samples to compare sleep spindles index (SSI). While to determine differences in duration, amplitude and intrinsic frequency; we also used Mann-Whitney U (independent samples) for each variable and for each registered brain region.

## RESULTS

### Sleep Spindle Index

**Figure 1:**
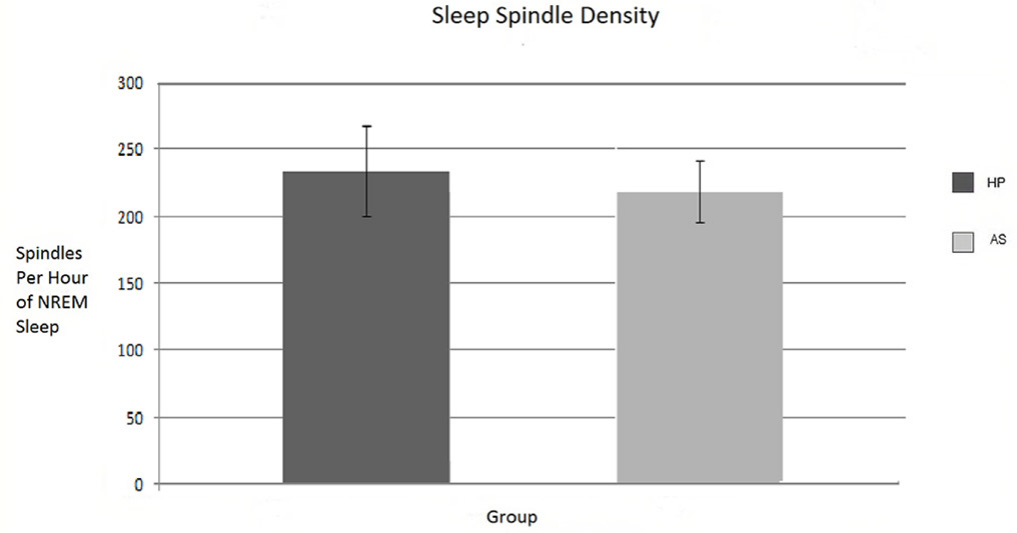
Number of spindles per hour of sleep in both groups. Note. Standard error marked on each bar. No signiﬁcant differences were observed between both groups (t test=-.559 and p=.5).

Healthy participants presented a spindle index of 234 spindles per hour of NREM, while AS group SSI value was 218 spindles per hour of NREM. However, this difference did not reach statistically significant levels (Figure 1).

### Sleep spindle duration

**Figure 2:**
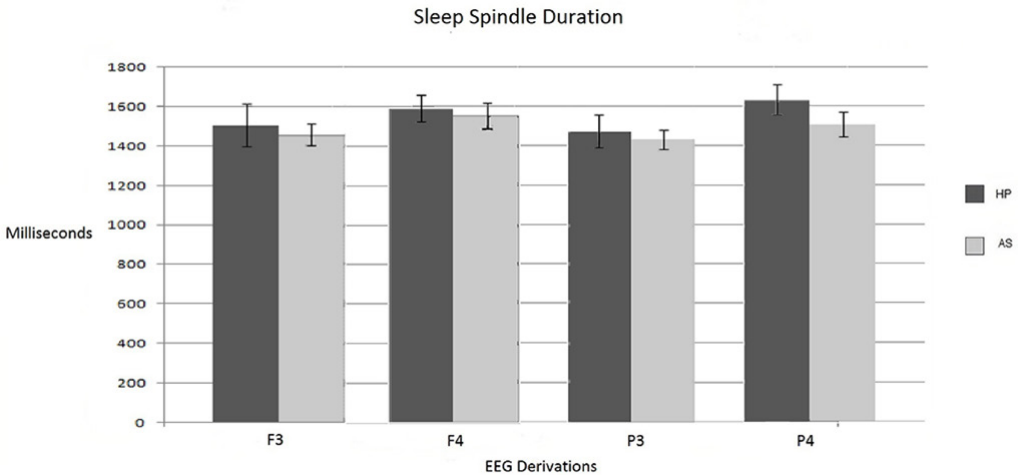
Average duration in milliseconds of the spindles analyzed in F3, F4, P3, and P4. Note: standard error marked on each bar.

Comparative analysis of the sleep spindles duration in leads F3, F4, P3, and P4 showed that values were always greater in the HP group. However, the observed differences were not statistically significant (U=32, *p*=.453; U=35, *p*=.627; U=36, *p*=.691 and U=19, *p*=.058, respectively) in none of the analyzed regions (Figure 2).

### Spindle amplitude

**Figure 3:**
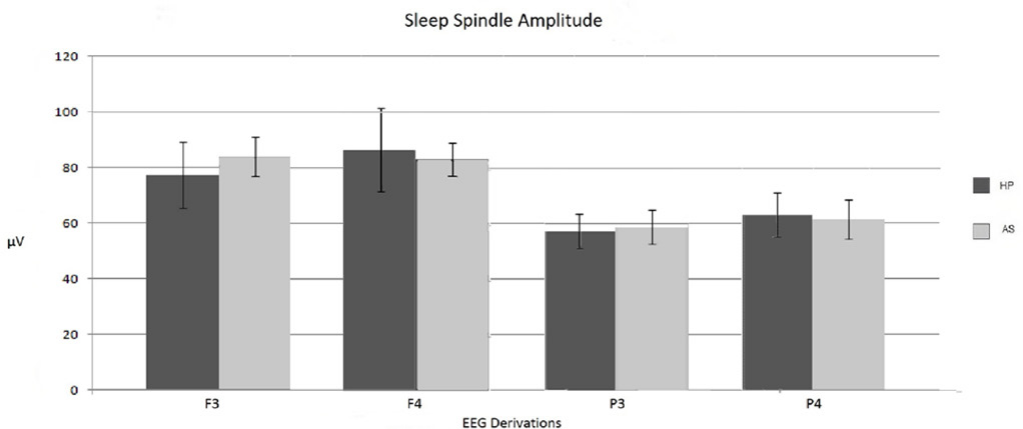
Average amplitude of sleep spindles in the different brain regions. Note: standard error marked on each bar. No signiﬁcant differences were found.

The maximum average amplitude reached by spindles in leads F3, F4, P3, and P4, did not show statistically significant differences between both groups (U=31, *p*=.402; U=39, *p*=.895; U=39, *p*=.895 and U=33, *p*=.508, respectively) (Figure 3). However, it is important to highlight that in both groups the spindles amplitude is significantly greater in frontal leads compared to parietal leads.

### Sleep spindle intrinsic frequency

**Figure 4:**
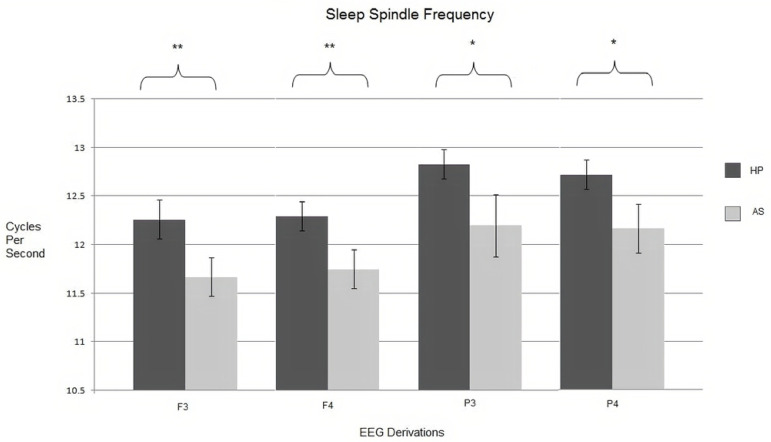
Sleep spindle intrinsic frequency registered in different electroencephalographic derivations. Note: standard error marked on each bar. Significant differences are observed: *p<.05, **p<.01.

The sleep spindle intrinsic frequency recorded in leads F3, F4, C3, and C4 was significantly lower in the AS group in all cases (U=10, *p*=.007; U=12, *p*=.012; U=18, *p*=.046 and U=15, *p*=.024) (Figure 4). All the results can be seen in Table 1.

**Table 1.** Sleep spindle values for both groups .* Differences statistically significant considering a level of significance of 0.05 ** Differences statistically significant considering a level of significance of 0.01.

	Group	Mean	SD	U	Sig
SSI	HP	234.23	69.78	39	.928
	AS	218.95	46.04		
Spindle Duration F3	HP	1504.67	218.59	32	.453
	AS	1458.23	104.82		
Spindle Duration F4	HP	1590.28	139.06	35	.627
	AS	1553.13	133.63		
Spindle Duration P3	HP	1474.26	168.21	36	.691
	AS	1433.34	99.46		
Spindle Duration P4	HP	1631.71	152.32	19	.058
	AS	1505.17	123.72		
Spindle Amplitude F3	HP	77.33	24.34	31	.402
	AS	84.13	15.27		
Spindle Amplitude F4	HP	86.75	31.17	39	.895
	AS	83.24	16.41		
Spindle Amplitude P3	HP	57.43	13.82	39	..895
	AS	58.93	12.91		
Spindle Amplitude P4	HP	63.11	16.74	33	.508
	AS	61.42	15.05		
Spindle Frequency F3	HP	12.26	.43	10	.007**
	AS	11.67	.40		
Spindle Frequency F4	HP	12.29	.29	12	.012**
	AS	11.75	.40		
Spindle Frequency P3	HP	12.83	.30	18	.046*
	AS	12.20	.65		
Spindle Frequency P4	HP	12.72	.52	15	.024*
	AS	12.17	.31		

## DISCUSSION AND CONCLUSION

After analyzing the sleep spindles of both groups, we observed spindles in a range of 9 to 14Hz, this frequency range is similar to that found by Clawson et al.^2^ (9-16Hz), the difference between both ranges may be due to the age of the participants in each study. However, unlike this study, we did not analyze alpha rhythm since our objective was only to analyze sleep spindles found in phase N2.

We found that the participants in the HP group show significantly faster sleep spindles in the frontal and parietal areas of the brain compared to the participants in the AS group. The comparative analysis of spindle amplitude displayed by the two groups did not show significant differences in any derivations; the values of spindle amplitude we observed are similar to those reported by other authors in healthy children^4,5,27,34^. However, it is interesting to outstand that frontal derivations (F3 and F4) of both groups of participants show spindle amplitude significantly higher than parietal derivations (P3 and P4). Regarding the sleep spindle duration, no significant differences were found either.

There are relatively few studies looking at the characteristics of sleep spindles in children with AS specifically. Unfortunately, the results present in this study are not consistent with those reported by Tessier et al.^29^, who found a lower density of sleep spindles in lead Fp2 and shorter duration of spindles in Fp1. These authors found lower intrinsic frequency in sleep spindles in the central region, while in the present investigation we found that the frequency of sleep spindles is lower in frontal areas, as well as in parietal areas. The observed differences may be due to the age and size of the samples studied, as well as in the EEG derivations analyzed^29^.

Studies of the brain activity’s ontogeny have shown that the sleep spindles intrinsic frequency increases gradually during the transition between childhood and adolescence concomitantly with brain maturation. After reaching adulthood, sleep spindle frequency remains unchanged^4^. The deficiencies in the development of gray and white matter present in patients with AS may affect the development of neural circuits responsible for the sleep spindle expression, which remain in stages prior to their age due to the immaturity of the systems that integrate the signals coming from thalamic reticular nuclei.

Some studies find a negative correlation between slow frontal spindles (<12Hz) and the IQ of children from 9 to 12 years of age, as well as a positive correlation between parietal fast spindles (>12Hz) and IQ. Among the tasks studied in these investigations are working memory, planning ability, and perceptual reasoning^2^. Other studies show that during the first hour of sleep a negative correlation is observed between the retention of information related to memorizing word pairs and the density of fast spindles^10^. The speed of information processing in children, defined as the time in which the participant solves a certain mental task, also has a positive correlation in relation to the density of slow spindles, while no correlation is observed with fast sleep spindles, this could be indicative of the relationship between slow sleep spindles and children’s cognitive development11. With this in mind, it is probable that the differences of sleep spindle’s intrinsic frequency found in the present study are related to the cognitive alterations observed in the neuropsychological development of children with Asperger’s syndrome^16-19^.

Slow and fast sleep spindles have topographical and functional differences. Slow spindles observed in frontal lobes are associated with frontal gyrus increased activity, while fast spindles observed in parietal lobes are associated with activity in areas involved with sensory-motor information processing35. Changes in spindle’s intrinsic frequency throughout the brain could alter function of the different areas of frontal and parietal lobes.

However, additional studies are required in order to understand the functional significance of sleep spindles on the cognitive processes of patients with AS.

As observed in schizophrenia, Down syndrome, and Alzheimer’s disease^12-14^, changes in the sleep microstructure of children with Asperger’s syndrome can lead to their use as an indicator of risk factors for the presence of neuropsychiatric disorders.

### Strengths and limitations of the study

The main strength of this study is the sample selection. In past research, participants with different diagnoses within the autism spectrum and of a wide variety of ages were used, while in this research a homogeneous sample was selected in terms of diagnosis and age. In addition, having a previous night of study guaranteed a better adaptation to the laboratory conditions, which helped to observe a more stabilized sleep.

As for the limitations of the study, the study had a small sample; future studies should consider expanding the sample. Unfortunately, it was not possible to control for the wake-sleep rhythm in the days preceding the PSG recordings. Also, since the sleep spindle identification was only visual, it was not possible to differentiate between fast and slow sleep spindles.
